# Complete Response to Immunotherapy Combined With Chemotherapy in a Patient With Gynecological Mixed Cancer Mainly Composed of Small Cell Neuroendocrine Carcinoma With High Tumor Mutational Burden: A Case Report

**DOI:** 10.3389/fonc.2022.750970

**Published:** 2022-06-20

**Authors:** Xingyun Su, Xinhui Zhou, Cheng Xiao, Wei Peng, Qiangfeng Wang, Yulong Zheng

**Affiliations:** ^1^ Department of Medical Oncology, The First Affiliated Hospital, Zhejiang University School of Medicine, Hangzhou, China; ^2^ Department of Gynecology, The First Affiliated Hospital, Zhejiang University School of Medicine, Hangzhou, China; ^3^ Department of Pathology, The First Affiliated Hospital, Zhejiang University School of Medicine, Hangzhou, China

**Keywords:** immune checkpoint inhibitor, gynecological tumor, small cell neuroendocrine carcinoma, tumor mutational burden, mixed cancer

## Abstract

Small cell neuroendocrine carcinoma (SCNEC) is rare in the gynecologic tract, which has high invasive and metastatic ability. Due to the aggressive behavior and lack of treatment, patients have an extremely poor prognosis. Here we report a 66-year-old female diagnosed with SCNEC in the gynecologic tract, mixed with endometrioid adenocarcinoma, squamous cell, and adenosquamous carcinoma. A tumor mutational burden of 13.14 Muts/Mb was detected by next-generation sequencing. The patient underwent a palliative operation of total hysterectomy with bilateral adnexectomy but suffered from disease progression in a short time after the operation. Chemotherapy (paclitaxel + carboplatin) combined with immunotherapy (toripalimab) was conducted every 3 weeks, achieving a partial response after 2 cycles of treatment. After 5 cycles of combined treatment, the patient consolidated with monotherapy of toripalimab for about half a year and achieved a complete response. Until December 2021, the patient has achieved 27 months of progression-free survival and maintains a continued complete response. This case is presented due to the rare combination of pathological types and durable response to treatment especially immunotherapy, suggesting the potential value of immunotherapy in SCNEC of the gynecologic tract.

## Introduction

Neuroendocrine carcinomas (NECs) are rare and aggressive malignancies categorized as low- and high-grade by the World Health Organization (1). High-grade NECs are further classified as small cell and large cell (1). Small cell neuroendocrine carcinoma (SCNEC) is the most common and poorly differentiated subtype, predominantly occurring in the lung and occasionally in the digestive and gynecologic tract (1). However, SCNEC constitutes only 2% of gynecological malignancies and usually occurs in the cervix and ovary (1). SCNEC in the gynecologic tract (SCNEC-GT) has a high incidence of lymphatic and distant metastasis with adverse outcomes (1, 2). The treatment of SCNEC-GT often includes surgery, radiation, and platinum-based chemotherapy, but no standard guideline has been achieved due to its rarity (1–3). Here we reported a 66-year-old female diagnosed with SCNEC-GT, mixed with endometrioid adenocarcinoma, squamous cell, and adenosquamous carcinoma. Until December 2021, the patient has achieved more than 27 months of progression-free survival (PFS) and has a continued complete response after the treatment consisting of surgery, chemotherapy, and immunotherapy. This case is presented due to the rare combination of four pathological types and durable response to treatment, especially immunotherapy.

## Case Presentation

**Figure 1:**
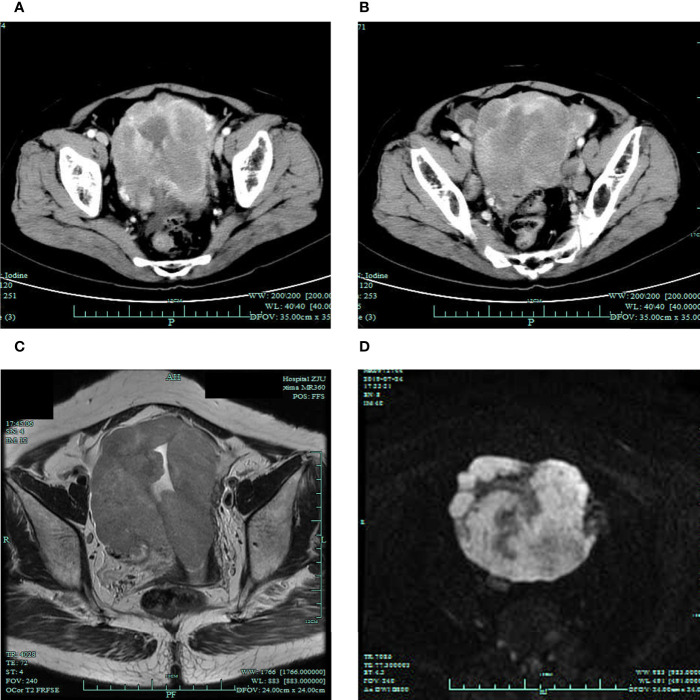
Radiographic findings before the treatment. **(A, B)** Preoperative CT scan showing the masses in the uterus and bilateral ovaries. MRI T2WI **(C)** and DWI **(D)** showing the mass in the uterus.

A 66-year-old Chinese woman was admitted to our hospital in July 2019, with a chief complaint of postmenopausal vaginal bleeding along with abdominal distension. During the physical examination, the uterus was enlarged, like that of a 4-month pregnancy, and with a lightly pressing pain. History of other diseases, family history of cancer, and habits of smoking or drinking were denied. No abnormality was found in the serum tumor markers (CEA, CA199, CA125, CA153, and AFP) or reproductive hormones (testosterone, estradiol, progesterone, prolactin, follicle stimulating hormone, and luteinizing hormone). The abdominal CT scan showed an irregular and uneven mass (92 × 93 mm) spreading from the uterine fundus to the cervix with inhomogeneous enhancement (**Figure 1A**). Besides this, masses with enhancement were found in the bilateral ovaries (left 20 x 15mm, right 24 × 25 mm) (**Figure 1B**). MRI showed that the mass in the uterus had an equal signal in T1WI, mixed (high or equal) signal in T2WI, and obviously high signal in DWI (**Figures 1C, D**).

**Figure 2:**
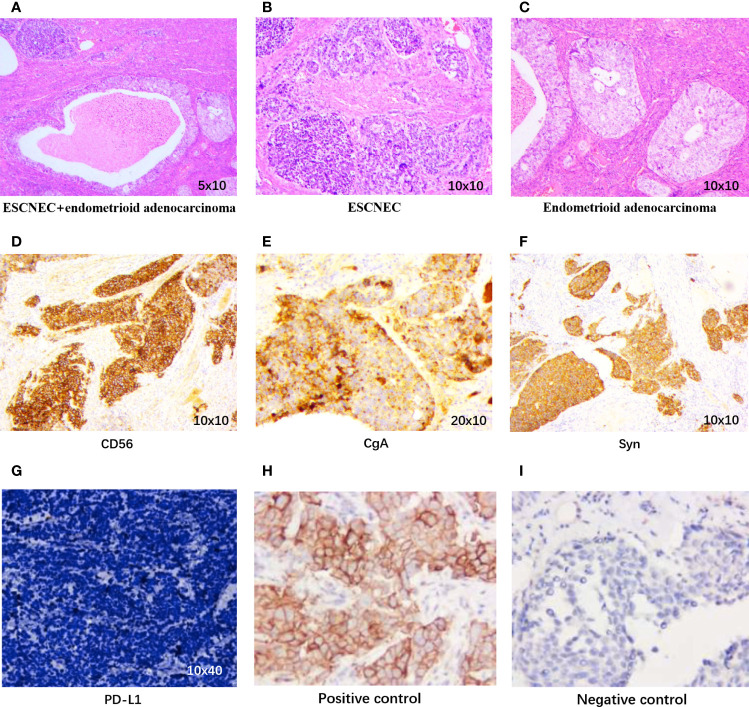
Pathological findings. **(A)** Coexistence of small cell neuroendocrine carcinoma (SCNEC) and endometrioid adenocarcinoma in the uterus (H&E, 5 × 10). **(B)** The SCNEC area shows small round cells in nests and clusters (H&E, 10 × 10). **(C)** Typical features of endometrioid adenocarcinoma (H&E, 10 × 10). Immunohistochemistry staining positive for **(D)** CD56 (10 × 10), **(E)** CgA (20 × 10), and **(F)** Syn (10 × 10). **(G)** PD-L1 expression of our case, **(H)** Positive control of PD-L1, **(I)** Negative control of PD-L1.

The patient underwent palliative operation of total hysterectomy with bilateral adnexectomy on July 26, 2019. The postoperative pathology showed that a small cell, high-grade NEC infiltrated the entire layers of the uterus, with a positive incisal margin in the left parametrium. The bilateral ovaries and right fallopian tube were also invaded by NEC with lymphovascular invasion. In addition, moderate–poor differentiated endometrioid adenocarcinoma and adenosquamous cancer were respectively detected in the right fallopian tube and the cervix. A nodule in the mesentery was confirmed as a metastasis of NEC combined with high–moderate differentiated squamous cell cancer. Besides this, atypical cells with increased nucleoplasm ratio indicating NEC were found in ascites. Based on postoperative pathology, the primary site of the tumor may be the uterus, and the tumor was stage IV according to the International Federation of Gynecology and Obstetrics (FIGO) staging (4). The expression of programmed cell death ligand 1 (PD-L1) was negative, with a tumor proportion score of less than 1%. The expression of p16 was positive. The pathological results are presented in **Figure 2**. Next-generation sequencing was conducted by a panel, including 457 cancer-associated genes for the tumor tissue in the uterus. A total of 15 alterations, including 12 single-nucleotide polymorphisms and 3 insertion/deletion variations, were detected (**Supplementary Table S1**). The tumor mutational burden (TMB) was 13.14 mutations/mega-base (Muts/Mb). The microsatellite status was stable (MSS) without length variation in five microsatellite locations (BAT-25, BAT-26, NR-21, NR-24, and MONO-27), which was consistent with the results of immunohistochemistry.

**Figure 3:**
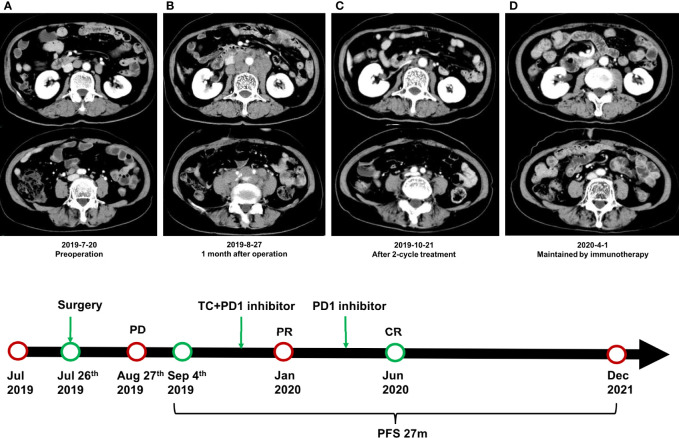
Radiographic findings during the treatment. **(A)** CT scan of the posterior peritoneum before surgery. **(B)** Multiple enlarged and merged lymph nodes were found in the posterior peritoneum at 1 month after surgery. **(C)** The lesions were obviously shrunk and evaluated as partial response after 2 cycles of combined treatment. **(D)** The disease achieved complete response during the consolidation treatment of toripalimab.

At 1 month after surgery (August 27), multiple enlarged and merged lymph nodes were found in the posterior peritoneum, surrounding aorta, and postcava and bilateral iliac vessels, which were not found in the CT scan before the operation (**Figures 3A, B**). Therefore, we regarded the patient as undergoing disease progression according to radiographic findings, although biopsy was not performed to confirm the pathology. Chemotherapy (paclitaxel, 300 mg d1 + carboplatin, 450 mg d1) combined with immunotherapy (toripalimab, 240 mg d1) was conducted every 3 weeks since September 4, 2019. After two cycles of treatment, the lesions were obviously shrunk and evaluated as partial response (PR) according to RECIST 1.1 standard (**Figure 3C**). However, the patient suffered from serious nausea and vomiting and was treated with a monotherapy of toripalimab for the 3rd cycle. For the 4th to 6th cycles, combined therapy was conducted again. After 6 cycles, the lesions were also evaluated as PR, and then a monotherapy of toripalimab was administrated every 3 weeks for consolidation after January 2020. During the consolidation treatment of immunotherapy, the lesions were continuously shrunk and achieved a complete response (CR) (**Figure 3D**). The consolidation treatment of toripalimab was terminated in June 2020 due to economic reasons, and a reexamination was conducted every 3 months. The latest CT scan was done in December 2021 at a local hospital, and it showed durable CR of disease. Until December 2021, the PFS of the patient has exceeded more than 27 months.

## Discussion

Collision and composite tumors are a coexistence of more than two distinct histological types, accounting for only 1–6% of gynecologic malignancies (5–7). The cause of collision and composite tumors has not been completely elucidated. The simultaneous exposure of a carcinogen to embryologically similar tissues may give rise to similar histological subtypes, but it cannot explain the tumors containing different pathological subtypes. Other factors such as viral infections or genetic mutations may also work. Alejo *et al.* found p16 to be expressed in up to 89% of NECs in the cervix, consistent with the results of human papillomavirus (HPV) detection (8). Besides this, the positive rate of HPV18 was higher in NECs than in other types of cervical cancer (8). Similar conclusions were drawn in a system review including 228 cases from 41 studies (9). Our case showed a very rare combination of four pathological subtypes, including SCNEC, endometrioid adenocarcinoma, squamous cell, and adenosquamous cancer. SCNEC was the major component spreading from the uterus to the fallopian tube, ovaries, and parametrium, but the origin of SCNEC, in our case, is inconclusive; it may be transformed from other histological types of cancer or arise independently. The p16 expression of our case was positive, given the association with HPV infection.

SCNEC-GTs are poorly differentiated and aggressive, commonly presenting as a huge bulky mass and with deep myometrial invasion at the time of diagnosis. Lymphovascular invasions are observed in the majority of cases, and distant metastases are also very common (1). Due to the early propensity for regional and systemic spread, patients have an extremely poor prognosis (1, 2). However, no standard guideline has been established due to the rarity of SCNEC-GT. The management of SCNEC is mainly based on retrospective studies of limited size and treatment experience of small cell lung cancer (SCLC) and conventional gynecological cancers (1). Besides this, the Hainsworth TEP regimen is recommended for carcinoma of unknown primary tumor site, which may be an alternative treatment strategy for SCNEC—especially for those that coexisted with different histological types (10). In recent years, the investigation of molecular and immune profile sheds light on cancer treatment, which is more important than histologic classification to guiding therapy (1, 11, 12).

The value of immune checkpoint inhibitors (ICIs) in NECs, especially in recurrent and progressive cases, has been brought into focus (13, 14). Wang *et al.* reported a case of a large cell NEC in the lung with high TMB, which achieved a durable response to pembrolizumab (15). Based on CheckMate-032, Impower-133, Keynote-028, and Keynote-158 trials, FDA approved nivolumab, atezolizumab, and pembrolizumab in the treatment of SCLC. Besides this, atezolizumab plus chemotherapy has been recommended for the first-line treatment of extensive SCLC (16). The activity of ICIs in NECs-GT is only reported in a few cases and mainly in the cervix. Paraghamian et al. used nivolumab in a patient with recurrent, metastatic, and PD-L1-negative cervix SCNEC experiencing a CR (17). Sharabi et al. reported a metastatic, chemotherapy-refractory cervix NEC case with a high TMB and treated with nivolumab combined with radiotherapy, which achieved CR for nearly more than 10 months (18). In our case, the combined treatment of chemotherapy and PD-1 inhibitor was conducted for 5 cycles, followed by a consolidation treatment of PD-1 inhibitor for about half a year. Until December 2021, our patient has achieved at least 27-month PFS, suggesting the potential value of ICIs in SCNEC-GT.

However, in a phase II study, no patient responded to the monotherapy of pembrolizumab, which was among seven women with small cell carcinoma (6 cervical and 1 vulvar) who were enrolled regardless of PD-L1 expression (19). Therefore, identifying patients who would probably benefit from ICIs is necessary in clinical practice. PD-L1 expression and MSI-High are generally accepted biomarkers for the response of ICIs (16, 20). Furthermore, previous studies provided mismatch repair-deficient as another biomarker (21), but NECs in the genital tract were overwhelmingly MSS and negative for PD-L1 expression (19, 22). Our case is also PD-L1 negative and MSS but has 13.14 Muts/Mb of TMB. A high TMB, generally regarded as a mutation load of more than 10 Muts/Mb, contributes to a high load of neoantigens which can be recognized by the immune system (23). Therefore, it is easier to eliminate tumor cells with TMB-H during immunotherapy. The Keynote-158 trial found an improved overall response rate in TMB-H patients treated with pembrolizumab in 10 types of cancer, including neuroendocrine tumor (24). Therefore, the FDA has approved pembrolizumab for adults and children with TMB-H solid tumors. Our patient experienced CR to PD-1 inhibitor and continuously benefited from immunotherapy, which may be explained by the high TMB.

The treatment duration of PD-1 inhibitor for our patients was about 9 months. In phase 3 clinical trials, the duration of immunotherapy usually ranges from up to 2 years to disease progression, but no firm conclusion has been achieved about the optional treatment duration. No significant difference was found in the overall survival (OS) of non-small cell lung cancer (NSCLC) patients treated with PD-1 inhibitor either for 2 years or until disease progression (25, 26). Antonia et al. suggested limiting the treatment to 1 year since stage III NSCLC patients benefit from a 1-year consolidation treatment of durvalumab after a concurrent chemoradiotherapy (27), but the Checkmate 153 trial showed that continuous nivolumab prolonged the PFS and OS of metastatic NSCLC patients in comparison with 1-year fixed duration (28). Therefore, whether a longer treatment may attribute to a better outcome is still uncertain. The investigation on the optimal treatment duration of ICIs is a new challenge to clinical practice. Previous researchers found that depth of response might be related to prognosis, and CR on ICIs helped in making the decision to stop the treatment (29).

Our patient experienced PR after the combination treatment and further achieved CR during the consolidation treatment of immunotherapy. Besides this, the patient had progression-free status for about 18 months after the termination of immunotherapy. The continuous antitumor effect after stopping immunotherapy may be attributed to the formation of immunological memory T cells (30). In the OAK trial, among 119 NSCLC patients treated with atezolizumab who lived for more than 24 months after randomization, 21% patients had progression disease as best response and 40% patients were PD-L1 negative, indicating that long-term survival was not limited to the responders (31). In the CA209-003 study, only 56% NSCLC patients who survived to 5 years with nivolumab completed the maximum treatment cycles (25). A durable response was also observed in Checkmate 017 and Checkmate 057 studies (32, 33). These results suggest that the survival benefit of ICIs might not only be correlated with treatment duration.

## Concluding Remarks

We reported a very rare combination of four pathological subtypes including SCNEC, endometrioid adenocarcinoma, squamous cell, and adenosquamous cancer. The patient was TMB-H and achieved more than 27 months of PFS after a treatment of surgery, chemotherapy, and immunotherapy. The durable response to PD-1 inhibitor indicates the potential value of immunotherapy in SCNEC-GT, and patients with TMB-H can be recommended to immunotherapy since it has been approved and they may achieve potential benefit.

## Data Availability Statement

The original contributions presented in the study are included in the article/**Supplementary Material**. Further inquiries can be directed to the corresponding author.

## Ethics Statement

Ethical approval was not required for this case report in accordance with the local legislation and institutional requirements. Written informed consent was obtained from the patient’s husband for the publication of any potentially identifiable images or data included in this article.

## Author Contributions

YZ contributed to the conception. XS contributed to the design and writing. YZ, XZ, and CX were involved in the care of the patient. WP contributed to the histopathological analysis and figure production. QW helped to revise the manuscript. All authors contributed to the article and approved the submitted version.

## Conflict of Interest

The authors declare that the research was conducted in the absence of any commercial or financial relationships that could be construed as a potential conflict of interest.

## Publisher’s Note

All claims expressed in this article are solely those of the authors and do not necessarily represent those of their affiliated organizations, or those of the publisher, the editors and the reviewers. Any product that may be evaluated in this article, or claim that may be made by its manufacturer, is not guaranteed or endorsed by the publisher.

## Abbreviations

SCNEC, small cell neuroendocrine carcinoma; NECs, neuroendocrine carcinomas; SCNEC-GT, small cell neuroendocrine carcinoma in gynecologic tract; PD-L1, programmed cell death ligand 1; TPS, tumor proportion score; NGS, next-generation sequencing; TMB, tumor mutational burden; Muts/Mb, mutations/mega-base; MSS, microsatellite status was stable; dMMR, mismatch repair-deficient; PR, partial response; CR, complete response; SCLC, small cell lung cancer; NSCLC, non-small cell lung cancer; ICIs, immune checkpoint inhibitors; PFS, progression-free survival; OS, overall survival.
