# Supplementary material for: Complete Response to Immunotherapy Combined With Chemotherapy in a Patient With Gynecological Mixed Cancer Mainly Composed of Small Cell Neuroendocrine Carcinoma With High Tumor Mutational Burden: A Case Report
Source: Front Oncol. 2022 Jun 20;12:750970. doi: 10.3389/fonc.2022.750970 (PMC9251297; doi:10.3389/fonc.2022.750970)
Supplement: Supplementary file 1 [file Table_1.docx]

**Supplementary Table 1** Genomic alterations identified by next generation sequencing

| **Single Nucleotide Polymorphisms** | | | | | |
| --- | --- | --- | --- | --- | --- |
| **Gene** | **Transcription** | **Exon** | **CDS mutation** | **Amino acid alteration** | **Mutation abundance** |
| CD274 | NM_014143 | 3 | c.329G>C | p.G110A | 12.38% |
| EMSY | NM_020193 | 19 | c.3068A>G | p.H1023R | 7.68% |
| EPPK1 | NM_031308 | 2 | c.5021G>A | p.R1674Q | 20.71% |
| ERRFI1 | NM_018948 | 4 | c.818C>T | p.S273F | 5.73% |
| GATA4 | NM_002052 | 4 | c.848G>A | p.R283H | 29.35% |
| GNAQ | NM_002072 | 2 | c.254C>T | p.T85M | 4.32% |
| INSR | NM_000208 | 14 | c.2753C>T | p.P918L | 21.16% |
| NF1 | NM_001042492 | 53 | c.7817A>G | p.D2606G | 4.26% |
| TNFSF11 | NM_003701 | 5 | c.943G>A | p.D315N | 4.29% |
| TP53 | NM_000546 | 8 | c.916C>T | p.R306* | 48.37% |
| TP53 | NM_000546 | 7 | c.743G>A | p.R248Q | 35.08% |
| XPO1 | NM_003400 | 2 | c.57C>G | p.F19L | 16.37% |
| **Insertion/Deletion variations** | | | | | |
| **Gene** | **Transcription** | **Exon** | **CDS mutation** | **Amino acid alteration** | **Mutation abundance** |
| PIK3R1 | NM_181523 | 10 | c.1138_1140delTTA | p.L380del | 17.32% |
| PTEN | NM_000314 | 7 | c.723_743delinsG | p.F241Lfs*5 | 36.53% |
| RB1 | NM_000321 | 11 | c.1112delC | p.P371Hfs | 46.88% |

CDS, sequencing coding for amino acids in protein.
